# Chemokines (CCL3, CCL4, and CCL5) Inhibit ATP-Induced Release of IL-1*β* by Monocytic Cells

**DOI:** 10.1155/2017/1434872

**Published:** 2017-12-20

**Authors:** Anca-Laura Amati, Anna Zakrzewicz, Robin Siebers, Sigrid Wilker, Sarah Heldmann, Dariusz Zakrzewicz, Andreas Hecker, J. Michael McIntosh, Winfried Padberg, Veronika Grau

**Affiliations:** ^1^Laboratory of Experimental Surgery, Department of General and Thoracic Surgery, Justus-Liebig-University Giessen, Giessen, Germany; ^2^Department of Biochemistry, Faculty of Medicine, Universities of Giessen and Marburg Lung Center, Giessen, Germany; ^3^George E. Wahlen Veterans Affairs Medical Center, Salt Lake City, UT, USA; ^4^Department of Biology, University of Utah, Salt Lake City, UT, USA; ^5^Department of Psychiatry, University of Utah, Salt Lake City, UT, USA; ^6^Excellence Cluster Cardiopulmonary System (ECCPS), German Center for Lung Research (DZL), Giessen, Germany

## Abstract

Chemokines and ATP are among the mediators of inflammatory sites that can enter the circulation via damaged blood vessels. The main function of chemokines is leukocyte mobilization, and ATP typically triggers inflammasome assembly. IL-1*β*, a potent inflammasome-dependent cytokine of innate immunity, is essential for pathogen defense. However, excessive IL-1*β* may cause life-threatening systemic inflammation. Here, we hypothesize that chemokines control ATP-dependent secretion of monocytic IL-1*β*. Lipopolysaccharide-primed human monocytic U937 cells were stimulated with the P2X7 agonist BzATP for 30 min to induce IL-1*β* release. CCL3, CCL4, and CCL5 dose dependently inhibited BzATP-stimulated release of IL-1*β*, whereas CXCL16 was ineffective. The effect of CCL3 was confirmed for primary mononuclear leukocytes. It was blunted after silencing CCR1 or calcium-independent phospholipase A2 (iPLA2) by siRNA and was sensitive to antagonists of nicotinic acetylcholine receptors containing subunits *α*7 and *α*9. U937 cells secreted small factors in response to CCL3 that mediated the inhibition of IL-1*β* release. We suggest that CCL chemokines inhibit ATP-induced release of IL-1*β* from U937 cells by a triple-membrane-passing mechanism involving CCR, iPLA2, release of small mediators, and nicotinic acetylcholine receptor subunits *α*7 and *α*9. We speculate that whenever chemokines and ATP enter the circulation concomitantly, systemic release of IL-1*β* is minimized.

## 1. Introduction

The orchestration of inflammation in response to tissue injury is essential for patient survival. Trauma-associated inflammation is a complex process involving release of danger-associated molecular patterns (DAMP), cell signaling, production and secretion of inflammatory mediators, local regulation of barrier functions, and recruitment of inflammatory cells [1–3]. Recruited leukocytes are essential for the elimination of cellular debris and foreign matter, for the prevention of infections, and for the initiation of tissue regeneration [1–3]. The site of inflammation should, however, communicate with the immune system with utmost caution, as exuberant inflammation carries the risk of inducing systemic inflammatory response syndrome (SIRS) that predisposes to life-threatening complications including systemic barrier dysfunction, sepsis, and multiorgan failure [1–3].

Countless mediators from wounds or other inflammatory sites enter the circulation via damaged blood vessels. The earliest among them is extracellular ATP and chemokines [4, 5]. Extracellular ATP, mainly originating from the cytoplasm of injured cells, is a DAMP that upon binding to receptor P2X7 expressed by primed monocytes/macrophages induces the assembly of the NLRP3 (NACHT, LRR, and PYD domain-containing protein 3) inflammasome [6–8]. NLRP3 inflammasomes activate the protease caspase-1 that enables maturation and release of inflammasome-dependent cytokines such as IL-1*β* and IL-18 [6–8]. IL-1*β* is a potent proinflammatory cytokine, predominantly but not exclusively produced by monocytes and macrophages, that plays a central role in host defense against viral, bacterial, and fungal infections [9]. High systemic levels of IL-1*β*, however, can cause severe systemic inflammation resembling SIRS [10]. Inflammasome activation also induces pyroptosis in monocytes/macrophages, a proinflammatory form of programmed cell death [11].

Chemokines are a family of predominantly proinflammatory peptides produced and released by immune and diverse nonimmune cells. Some chemokines are constitutively expressed and mainly regulate steady-state trafficking of leukocytes, while the majority are induced by DAMP or pathogen-associated molecular patterns (PAMP) [12]. Inducible chemokines primarily activate and attract leukocytes to the site of inflammation, and the spectrum of different chemokines that are released determines the cellular composition of the infiltrate. Each leukocyte subpopulation expresses a characteristic set of chemokine receptors that belong to the family of G protein-coupled receptors [13]. Monocytes are mainly mobilized by CC-chemokines that bind to CC-receptors (CCR), whereas they normally do not respond to CXC-chemokines as they are lacking their cognate CXC-receptors (CXCR) [12, 14, 15].

When chemokines that attract monocytes enter the circulation together with ATP, we predict a prioritization of the chemotactic signal that enables cellular infiltration of the inflamed tissue but prevents IL-1*β* release to avoid SIRS. Hence, monocytes should be attracted to the site of inflammation, whereas ATP-induced release of inflammasome-dependent cytokines into the blood stream and premature pyroptosis of monocytes should be controlled. This prediction prompted us to test the hypothesis that CCR activation down-modulates ATP-dependent release of IL-1*β* by lipopolysaccharide- (LPS-) primed human monocytic U937 cells. In line with this hypothesis, we demonstrate that chemokines CCL3 (MIP1*α*), CCL4 (MIP1*β*), and CCL5 (RANTES) efficiently inhibit release of IL-1*β* in response to ATP receptor stimulation. We provide evidence that the effect of CCL3 is mediated via CCR1 and calcium-independent phospholipase A2 (iPLA2), release of a soluble small mediator and nicotinic acetylcholine receptors (nAChR) containing subunits *α*7 and *α*9.

## 2. Material and Methods

### 2.1. U937 Cells

The human histiocytic lymphoma cell line U937 was obtained from the German Collection of Microorganisms and Cell Cultures (Braunschweig, Germany) and cultured in RPMI 1640 (Gibco by Life Technologies, Darmstadt, Germany), supplemented with 10% fetal calf serum (Biochrome, Berlin, Germany), and 2 mM L-glutamine (Gibco), at 37°C, 5% CO_2_. Experiments were carried out in 24-well plates containing 10^6^ cells in 1 ml. Cells were primed for 5 h with 1 *μ*g/ml LPS from *Escherichia coli* (L2654; Sigma-Aldrich, Taufkirchen, Germany) followed by stimulation with the P2X7 receptor agonist BzATP (2′(3′)-O-(4-benzoyl-benzoyl) ATP triethylammonium salt (Sigma-Aldrich or Jena Bioscience, Jena, Germany; 100 *μ*M). Cell culture supernatants were harvested 30 min later and stored at −20°C before detection of IL-1*β* by Quantikine® Immunoassay (R&D Systems, Minneapolis, MN) and measurement of lactate dehydrogenase (LDH) using the CytoTox 96® Non-Radioactive Cytotoxicity Assay (Promega, Madison, WI).

Different concentrations of chemokines CCL3, CCL4, CCL5, or CXCL16 (R&D Systems; 0.1–50 ng/ml) were applied together with BzATP, in the presence or absence of antagonists mecamylamine hydrochloride (100 *μ*M; Sigma-Aldrich), *α*-bungarotoxin (1 *μ*M; Tocris Bioscience, Bristol, UK), and strychnine hydrochloride (10 *μ*M; Sigma-Aldrich) or of the conotoxins ArIB [V11L,V16D] (500 nM) or RgIA4 (200 nM) [16–19]. To inhibit phospholipase A2 (PLA2), 50 *μ*M arachidonyl trifluoromethyl ketone (ATK; Enzo Life Sciences, Lausen, Switzerland) or 50 *μ*M bromoenol lactone (BEL; Enzo Life Sciences) was used. In some experiments, acetylcholine (Sigma-Aldrich; 10 *μ*M) was included as a positive control.

### 2.2. Human PBMC

Studies on human blood from male healthy nonsmoking volunteers were approved by the local ethics committee of the University of Giessen (number 81/13). PBMC were freshly isolated from heparinized blood by Leucosep gradients (Greiner Bio-One, Frickenhausen, Germany). In IL-1*β* release experiments, cells were pulsed with 0.5 ng LPS/ml before separation and PBMC were cultured for 3 h in RPMI 1640, 10% FCS, 2 mM L-glutamine. Nonadherent cells were removed, cell culture medium was replaced, and BzATP (100 *μ*M) was added for 30 min in the presence or absence of CCL3 (10 ng/ml). IL-1*β* and LDH was measured in cell culture supernatants.

### 2.3. Gene Silencing

U937 cells were transfected with *CCR1* or *PLA2G6* human siRNA ON-TARGETplus SMARTpool (GE Dharmacon, Lafayette, CO, USA), at a concentration of 30 pmol siRNA per 1 × 10^6^ cells, using the Amaxa® Cell Line Nucleofector® Kit C from Lonza (Cologne, Germany). ON-TARGETplus Nontargeting Control Pool (GE, Dharmacon) was included as a negative control. The cells were incubated under standard conditions for 48 hours before IL-1*β* release studies were performed. The efficiency of gene silencing was analyzed by real-time RT-PCR (*CCR1* and *PLA2G6*) and Western blotting (iPLA2). The protein expression of CCR1 by U937 cells was below the threshold of detection in both Western blotting and flow cytometry.

### 2.4. RNA Isolation and Real-Time RT-PCR

RNA was isolated from untreated and LPS-primed U937 cells as well as from siRNA-treated U937 cells at the end of the IL-1*β* release experiment. Total RNA extracted using the Qiagen RNeasy Miniprep Kit (Qiagen, Hilden, Germany) and cDNA was produced using M-MLV reverse transcriptase (Promega, Mannheim, Germany). Real-time PCR was performed in an ABI 7700 Sequence Detection System (PE Applied Biosystems, Foster City, CA) using Platinum SYBR green qPCR Super Mix-UDG (Invitrogen). The expression of hydroxymethylbilane synthase (*HMBS*; alias porphobilinogen deaminase, *PBGD*) was analyzed for data normalization. All primers were synthetized by Eurofins Genomics (Ebersberg, Germany). Primer sequences for chemokine receptors are indicated in Table 1; primers for *HMBS* were published before [20]. To validate the primer pairs, RNA from freshly isolated human peripheral blood mononuclear cells (PBMC) was included as a positive control. In negative controls, the cDNA was replaced by water.

PCR products were separated in 1.5% agarose gels together with GeneRuler™ 100 bp DNA Ladder (Thermo Fischer Scientific, Darmstadt, Germany) to assess their size. To further confirm the identity of the amplicons, DNA bands were excised from the gel, eluted (MinElute® PCR Purification Kit, Qiagen), and sequenced by SeqLab (Göttingen, Germany). Data were analyzed using the 2^ΔCt^ method, where ΔCt is the difference between the Ct of the housekeeping gene and of the gene of interest, and the mean values of the siRNA controls were set to one arbitrary unit.

### 2.5. Conditioned Medium

U937 cells were primed with LPS in RPMI 1640 as described but in the absence of fetal calf serum. Thereafter, CCL3 (10 ng/ml) was added for 30 min and cell-free cell culture supernatants were ultrafiltrated using Amicon® Ultra Centrifugal filters (Merck Millipore, Darmstadt, Germany) with a cut-off of 3 kDa. The efficiency of the ultrafiltration was controlled by SDS-gel electrophoresis followed by silver staining. The low molecular mass fraction was tested in IL-1*β* release experiments.

### 2.6. SDS-Gel Electrophoresis and Silver Staining

Different fractions of cell culture supernatant were resolved on 15% reducing SDS-polyacrylamide gels. The gels were stained with the Silver Stain Plus (Bio-Rad Laboratories, Munich, Germany) according to the manufacturer's instructions and documented using a gel imaging system (Intas, Göttingen, Germany).

### 2.7. Western Blotting

U937 cells were lysed, and proteins were separated on 10% SDS-polyacrylamide gels and transferred onto Immobilon polyvinylidene difluoride membranes (Millipore, Billerica, MA) together with dual color precision plus protein standards (Bio-Rad, Hercules, CA). Membranes were blocked with 5% BSA diluted in PBS and incubated with polyclonal rabbit antibodies to iPLA2 (1 : 5000; Sigma-Aldrich) or mouse monoclonal antibodies to *β*-actin (1 : 50000; Sigma-Aldrich). Bound primary antibodies were detected with secondary horse radish peroxidase-labeled antibodies (1 : 5000; Dako, Glostrup, Denmark), lumi-light substrate (Roche, Mannheim, Germany), and high-performance chemiluminescence films (GE Healthcare Bio-Sciences, Uppsala, Sweden).

### 2.8. Statistical Analyses

Results are presented as individual data points, median, and percentiles 25 and 75. Data were analyzed by the nonparametric Kruskal-Wallis test followed by the Mann–Whitney rank sum test using the SPSS software (Munich, Germany). *p* values below 0.05 were considered as statistically significant.

## 3. Results and Discussion

### 3.1. CCL-Induced Inhibition of IL-1*β* Release

**Figure 1:**
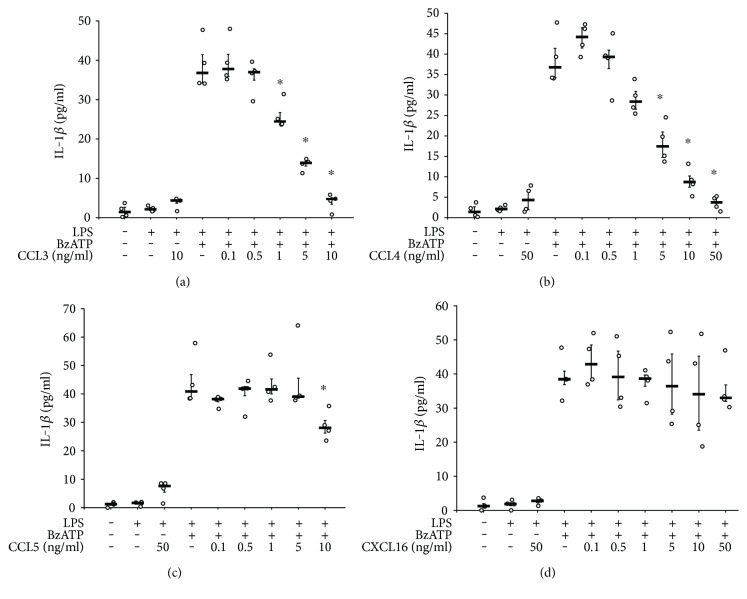
CCL chemokines inhibit ATP-induced IL-1*β* release. (a) Human monocytic U937 cells were primed with LPS (1 *μ*g/ml, 5 h) and activated with 2′(3′)-O-(4-benzoylbenzoyl)adenosine-5′-triphosphate (BzATP; 100 *μ*M, 30 min). The release of IL-1*β* to the cell culture supernatant was measured by ELISA. Chemokines CCL3 (a), CCL4 (b), and CCL5 (c) dose dependently and efficiently inhibited the BzATP-induced release of IL-1*β*. In contrast, chemokine CXCL16 (d) was ineffective. Data are presented as individual data points, bars indicate median, and whiskers percentiles are 25 and 75; *n* = 4; ^∗^*p* = 0.029, in comparison to cells treated with LPS and BzATP.

First experiments were performed to test our hypothesis that CCL chemokines that attract monocytes inhibit the ATP-dependent release of IL-1*β* by LPS-primed human monocytic U937 cells, whereas CXCL16, a chemokine that does not target monocytes [12], is ineffective. U937 cells were primed with LPS (1 *μ*g/ml) for 5 h followed by activation with the P2X7 agonist BzATP (100 *μ*M) for 30 min. In line with previous data [18, 20], LPS alone did not stimulate the release of IL-1*β* into the cell culture medium, whereas the consecutive application of LPS and BzATP induced its release (Figures 1(a), 1(b), 1(c), and 1(d)). In contrast, IL-18 was barely detectable (data not shown). In line with our hypothesis, the BzATP-induced release of IL-1*β* was dose dependently and efficiently inhibited by chemokines CCL3, CCL4, and CCL5 (*p* = 0.029; *n* = 4, each), whereas chemokine CXCL16 was ineffective (Figures 1(a), 1(b), 1(c), and 1(d)). When fully inhibitory concentrations of chemokines CCL3 (10 ng/ml), CCL4 (50 ng/ml), and CCL5 (50 ng/ml) were applied to LPS-primed U937 cells in the absence of BzATP, no IL-1*β* was released into the cell culture medium (Figures 1(a), 1(b), and 1(c)); similarly, 50 ng/ml CXCL16 had no effect (Figure 1(d)). The IC50 values were in the range of 1–5 ng/ml for CCL3 and CCL4 and about 10 ng/ml for CCL5. These IC50 values are in the range of the EC50 values typical for the activation of their cognate receptors. CCL3 was selected for the following elucidation of the signaling pathway.

**Figure 2:**
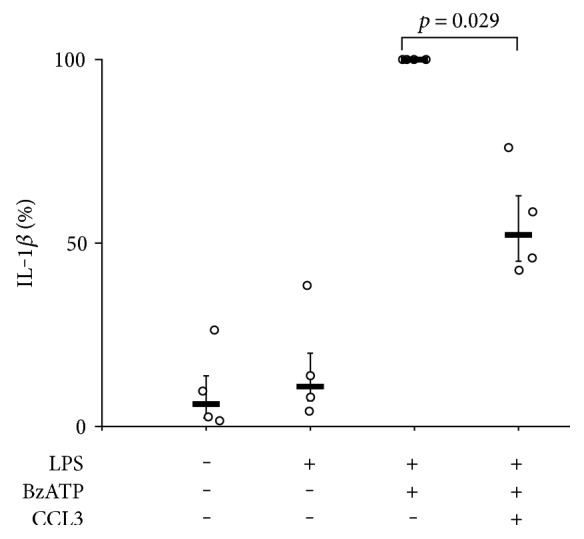
CCL3 inhibits ATP-induced IL-1*β* release by human peripheral blood mononuclear cells (PBMC). Blood from healthy volunteers was pulsed with 0.5 ng LPS/ml before purification of PBMC. PBMC were cultured for 3 h, and 2′(3′)-O-(4-benzoylbenzoyl)adenosine-5′-triphosphate (BzATP; 100 *μ*M) was added for 30 min in the presence or absence of CCL3 (10 ng/ml). IL-1*β* was measured in cell culture supernatants, and the values obtained in the supernatants of cells treated with LPS and BzATP were set to 100%. Data are presented as individual data points, bars indicate median, and whiskers percentiles are 25 and 75; *n* = 4.

The properties of cell lines typically differ considerably from primary cells. To test if the inhibitory effect of CCL3 on BzATP-mediated release of IL-1*β* also applies to primary monocytic cells, PBMC from healthy donors were investigated (Figure 2). Adherent LPS-pulsed PBMC secreted a small amount of IL-1*β* (median 344 ng/ml, range 247–468 ng/ml; *n* = 4) within 30 min, application of BzATP (100 *μ*M) increased the IL-1*β* release (median 3.6 pg/ml, range 0.6–9.2 pg/ml; *n* = 4), and CCL3 significantly reduced the release of IL-1*β* (median 2.2 pg/ml, range 0.4–4.2 pg/ml; *n* = 4).

### 3.2. Cell Viability

LDH concentrations in the cell culture supernatants of U937 cells and PBMC remained below 4% and 10%, respectively, of the total LDH of lysed cells, irrespective of the experimental setting. Only in experiments involving transfection of U937 cells with siRNA, up to 7% LDH release was measured (data not shown).

### 3.3. Signaling via CCR

**Figure 3:**
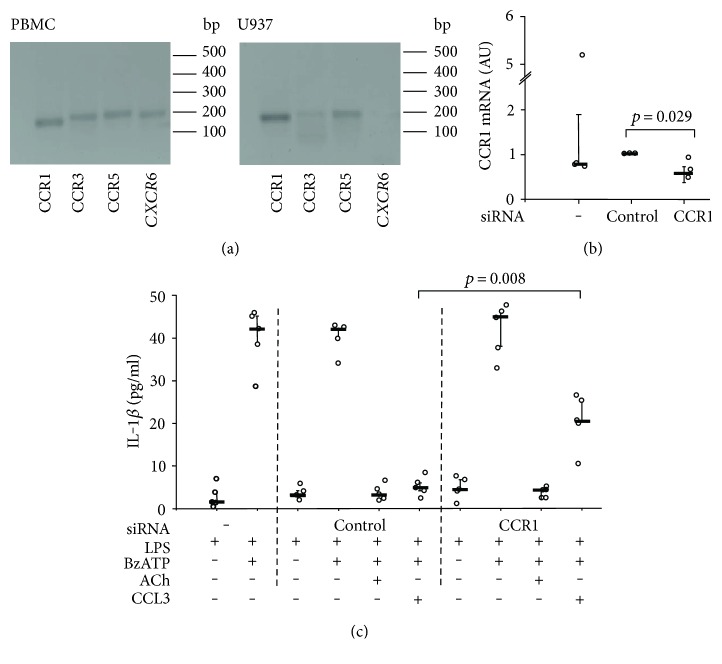
Signaling via chemokine receptor CCR1. (a) The mRNA expression of chemokine receptors *CCR1*, *CCR3*, *CCR5*, and *CXCR6* was investigated in U937 cells by real-time RT-PCR, and the amplicons were separated by gel electrophoreses together with a base pair (bp) ladder and detected by GelRed™ Nucleic Acid Dye. Peripheral blood mononuclear cells (PBMC) obtained from healthy volunteers served as positive control. (b, c) U937 cells were transfected with scrambled control siRNA or with siRNA targeting *CCR1*. These cells were primed with LPS (1 *μ*g/ml, 5 h) followed by activation with 2′(3′)-O-(4-benzoylbenzoyl)adenosine-5′-triphosphate (BzATP) (100 *μ*M, 30 min). (b) The efficiency of the siRNA transfection was verified by real-time RT-PCR. Values obtained for cells treated with siRNA targeting CCR1 were statistically compared to those transfected with control siRNA. (c) IL-1*β* was measured in the cell culture supernatant by ELISA. In untreated and in control-transfected cells, CCL3 (10 ng/ml) fully inhibited the BzATP-induced release of IL-1*β*, whereas silencing of *CCR1* significantly blunted the inhibitory effect of CCL3. In all experiments, acetylcholine (ACh; 10 *μ*M) was included as a positive control. Data are presented as individual data points, bars indicate median, and whiskers percentiles are 25 and 75; *n* = 5.

Chemokines CCL3, CCL4, and CCL5 are known agonists of chemokine receptors CCR1, CCR3, and CCR5 [12]. CXCL16 binds to receptor CXCR6 [12]. First, we analyzed by real-time RT-PCR if the mRNA of these chemokine receptors is expressed by U937 cells. We were indeed able to detect the mRNA for *CCR1, CCR3*, and *CCR5*, whereas no mRNA for *CXCR6* was detected after 45 cycles of amplification. In PBMC that were included as a positive control, the mRNA of all four chemokine receptors was expressed and no DNA was amplified in negative controls. DNA bands of the expected size were detected in agarose gels (Figure 3(a)), and DNA sequencing confirmed product identity.

To analyze if CCR are involved in the CCL3-induced control of IL-1*β* release, we transfected U937 cells with siRNA targeting *CCR1* gene expression and primed these cells with LPS. We selected *CCR1* for gene silencing because the mRNA of this receptor seemed to be more abundant in U937 cell than *CCR3* and *CCR5* mRNA. Control cells were transfected with irrelevant control siRNA. In contrast to control siRNA, siRNA targeting CCR1 significantly inhibited the mRNA expression (*p* = 0.029, *n* = 4; Figure 3(b)). Gene silencing did not change the BzATP-induced release of IL-1*β*, and transfection of control siRNA did not impair the inhibitory effect of CCL3 on IL-1*β* release (Figure 3(c)). Silencing of *CCR1*, however, significantly (*p* = 0.008, *n* = 5, each) blunted the inhibitory effect of CCL3 (Figure 3(c)). These results suggest that the anti-inflammatory effect of CCL3 is at least in part mediated by conventional chemokine receptors. The effect of CCL3 was not fully inhibited by silencing of *CCR1*. This observation may be explained by the fact that siRNA-treatment did not fully abolish the expression of *CCR1* and that apart from CCR1 other chemokine receptors such as CCR3 and CCR5 are involved.

### 3.4. Involvement of nAChR

**Figure 4:**
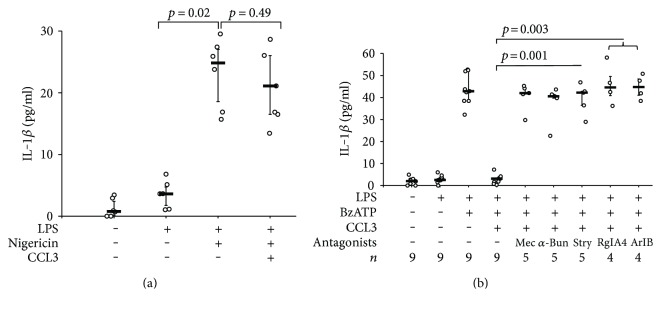
CCL3 signaling involves nicotinic acetylcholine receptors. Human monocytic U937 cells were primed with LPS (1 *μ*g/ml, 5 h) and activated (a) with the pore-forming bacterial toxin nigericin (50 *μ*M) or (b) with 2′(3′)-O-(4-benzoylbenzoyl)adenosine-5′-triphosphate (BzATP; 100 *μ*M, 30 min). The release of IL-1*β* to the cell culture supernatant was measured by ELISA. (a) Nigericin induced the release of IL-1*β* by LPS-primed U937 cells, but the chemokine CCL3 (10 ng/ml) did not impair the nigericin-triggered release of IL-1*β* (*n* = 6). (b) Nicotinic antagonists mecamylamine (Mec; 100 *μ*M), *α*-bungarotoxin (*α*-Bun; 1 *μ*M), and strychnine (Stry; 10 *μ*M), as well as *α*-conotoxins ArIB [V11L,V16D] (200 nM) and RgIA4 (200 nM), reversed the inhibitory effect of CCL3. Data are presented as individual data points, bars indicate median, and whiskers percentiles are 25 and 75.

Recently, we discovered that stimulation of nAChR containing subunits *α*7, *α*9, and *α*10 efficiently inhibits the BzATP-triggered release of IL-1*β* by primary human monocytes and by LPS-primed U937 cells [18, 20]. This pathway is specific for ATP-dependent inflammasome activation as it inhibits the ion channel function of receptor P2X7. This prompted us to test if CCL3 also inhibits the ATP-independent IL-1*β* release induced by the pore-forming bacterial toxin nigericin (50 *μ*M). In this experimental setting, we added apyrase (0.5 U/ml), an enzyme that efficiently cleaves endogenous ATP that might be released by LPS-primed and nigericin-treated U937 cells. Stimulation of LPS-primed U937 cells with nigericin/apyrase for 30 min induced IL-1*β* secretion as described before (Figure 4(a)) [20]. Of note, nigericin-induced secretion of IL-1*β* was not reduced by CCL3 (Figure 4(a)). This result is in line with our assumption that CCL3 triggers a mechanism that is specific for ATP-dependent inflammasome activation.

In the next set of experiments, we tested the rather provocative hypothesis that nAChR are involved in CCL3 signaling using a panel of nAChR antagonists that do not induce the release of IL-1*β* when applied alone [18, 20] (Figure 4(b)). The inhibitory effect of CCL3 on BzATP-induced release of IL-1*β* by U937 cells was indeed sensitive to mecamylamine (100 *μ*M; *p* = 0.001, *n* = 5 versus *n* = 9), a general nAChR antagonist. Similarly, *α*-bungarotoxin (1 *μ*M; *p* = 0.001, *n* = 5 versus *n* = 9) and strychnine (10 *μ*M; *p* = 0.001, *n* = 5 versus *n* = 9), reagents that preferentially antagonize nAChR containing subunits *α*7 and *α*9, were efficient (Figure 4(b)). To differentiate between those subunits, we made use of *α*-conotoxins ArIB [V11L,V16D] (500 nM), specific for nAChR containing an *α*7 subunit, and RgIA4 (200 nM), an antagonist of *α*9*α*10 nAChR [16–19]. Both *α*-conotoxins antagonized the effect of CCL3 (*p* = 0.003, *n* = 5 versus *n* = 9), suggesting that signal transduction involves nAChR subunits *α*7 and *α*9 (Figure 4(b)). These nAChR subunits previously have been shown to be expressed by U937 cells and by human monocytes [20, 21].

These results are in line with the idea that chemokine signaling triggers a recently described mechanism involving activation of metabotropic functions of noncanonical nAChR containing subunits *α*7 and *α*9 that inhibit P2X7 signaling and, consequently, BzATP-induced release of IL-1*β* [18, 20]. Of note, the inhibitory effect of acetylcholine (10 *μ*M) was not impaired by silencing of CCR expression (Figure 3(c)), suggesting that nAChR are acting downstream of CCR.

### 3.5. Release of Soluble Factors

We wanted to solve the question of how CCR activation links to cholinergic signaling. As at least some of the effective nicotinic antagonists such as *α*-bungarotoxin and the *α*-conotoxin RgIA4 probably do not enter the cytoplasm of the target cell due to their size and hydrophilicity, we assume that relevant nAChR are localized in the plasma membrane. Hence, we postulated the involvement of a soluble small nicotinic agonist that is released to the cell culture medium in response to CCL3.

**Figure 5:**
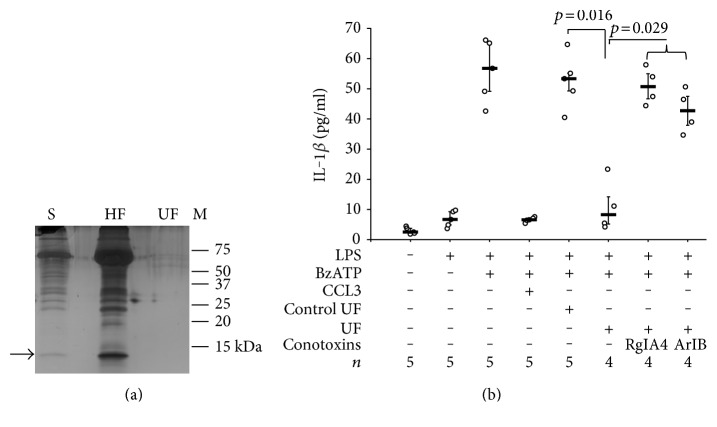
Release of small mediators in response to CCL3. U937 cells were primed with LPS (1 *μ*g/ml, 5 h) and activated with 2′(3′)-O-(4-benzoylbenzoyl)adenosine-5′-triphosphate (BzATP; 100 *μ*M, 30 min). The release of IL-1*β* to the cell culture supernatant was measured by ELISA. CCL3, which was included as a positive control, inhibited the BzATP-induced release of IL-1*β*. An ultrafiltrate (UF) was produced containing the low molecular mass fraction (<3 kDa) of the cell culture supernatant (S) of LPS-primed U937 cells treated with CCL3 (10 ng/ml) for 30 min. For the production of control UF, CCL3 was added to the cell-free supernatant of LPS-primed U937 cells shortly before ultrafiltration. (a) The UF and the high molecular mass fraction (HF) obtained by ultrafiltration were separated in a 15% SDS-polyacrylamide gel along with a molecular mass marker (M) followed by silver staining. The arrow is pointing to the band corresponding to CCL3. Proteins with higher molecular mass are bovine serum albumin (66.5 kDa) and its contaminations that were added to the CCL3 preparation for stabilization. One typical result out of 5 experiments is depicted. (b) Control UF had no effect on the BzATP-induced release of IL-1*β* by LPS-primed U937 cells, whereas UF significantly reduced the IL-1*β* release. The effect of the UF was sensitive to conotoxins ArIB [V11L,V16D] (200 nM) and RgIA4 (200 nM). Data are presented as individual data points, bars indicate median, and whiskers percentiles are 25 and 75.

To test this hypothesis, LPS-primed U937 cells were stimulated with CCL3, conditioned cell culture medium was harvested 30 min later, and a low molecular weight fraction of this medium devoid of CCL3 was produced by ultrafiltration (Figure 5(a)). As a control, conditioned medium was produced in the absence of CCL3, but CCL3 was added to the cell-free supernatant shortly before ultrafiltration. In line with our hypothesis, the ultrafiltrate of conditioned medium significantly inhibited the BzATP-stimulated release of IL-1*β* by LPS-primed U937 cells, whereas the ultrafiltrate of the control conditioned medium was ineffective (*p* = 0.016; *n* = 4 versus *n* = 5) (Figure 5(b)). We wondered if this small mediator acts as a nicotinic agonist at nAChR subunits and were indeed able to demonstrate that the effect of conditioned medium is antagonized by conotoxins ArIB [V11L,V16D] and RgIA4 (*p* = 0.029; *n* = 4 each) that are specific for nAChR subunits *α*7 and *α*9, respectively (Figure 5(b)). These results suggest that CCL3 induces the secretion of nicotinic agonists by U937 cells.

### 3.6. Involvement of iPLA2

**Figure 6:**
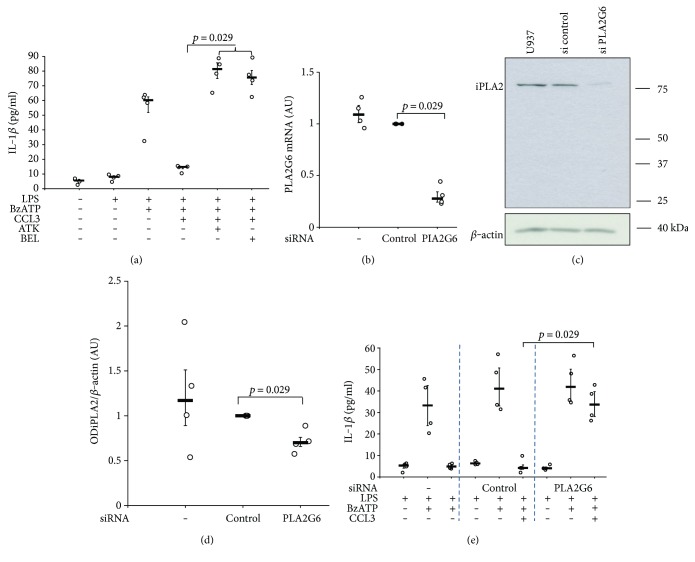
CCL3 signaling involves calcium-independent phospholipase A2 (iPLA2). Human monocytic U937 cells were primed with LPS (1 *μ*g/ml, 5 h) and activated 2′(3′)-O-(4-Benzoylbenzoyl)adenosine-5′-triphosphate (BzATP; 100 *μ*M, 30 min). The release of IL-1*β* to the cell culture supernatant was measured by ELISA. (a) CCL3 (10 ng/ml) inhibited the release of IL-1*β* in response to BzATP. The general PLA2 inhibitor arachidonyl trifluoromethyl ketone (ATK) and the specific iPLA2 inhibitor bromoenol lactone (BEL) reversed CCL3-dependent inhibition. (b–e) Expression of iPLA2 by U937 cells was silenced by siRNA (*PLA2G6*); scrambled siRNA served as control. Silencing of *PLA2G6* expression was efficient as revealed by real-time RT-PCR (b) and by Western blotting (c, d). Data in (b) and (d) are given as arbitrary units. (c, d) In Western blotting experiments, *β*-actin served as a loading control, the optical density (OD) of the immunopositive bands was measured, and the ratio of the OD of iPLA2 and *β*-actin was formed. (b, d) Values obtained for cells treated with siRNA targeting CCR1 were statistically compared to those transfected with control siRNA. (e) After treatment with siRNA targeting *PLA2G6* expression, the inhibitory effect of CCL3 on IL-1*β* release was blunted. In untreated control cells and upon transfection of control siRNA, CCL3 was effective. Data are presented as individual data points, bars indicate median, and whiskers percentiles are 25 and 75, *n* = 4, each.

How does chemokine signaling translate into the release of a bioactive factor? We reported recently that phosphocholine and phosphocholine-modified macromolecules are nicotinic agonists at noncanonical nAChR of human and mouse monocytes that control P2X7 receptor [18, 20]. Hence, it is conceivable that the bioactive factor is a metabolite of membrane phosphatidylcholines. Calcium-dependent phospholipase A2 (cPLA2) and iPLA2 use phosphatidylcholines as substrate to form arachidonic acid, a precursor of several lipid mediators, and lysophosphatidylcholines, a metabolite that can be further degraded to glycerophosphocholine, phosphocholine, or lysophosphatidic acid and choline [22, 23]. Interestingly, iPLA2 is essential for the chemotactic response to CCL2/MCP1 [24, 25]. Therefore, we investigated if PLA2 is involved in CCL3 signaling using ATK (50 *μ*M), an inhibitor of cPLA2 and iPLA2. ATK enabled BzATP-induced IL-1*β* release in despite of the presence of CCL3 (*p* = 0.029, *n* = 4; Figure 6(a)). BEL (50 *μ*M), a specific inhibitor of iPLA2, was also effective, suggesting that iPLA2 plays an essential role (*p* = 0.029, *n* = 4; Figure 6(a)). The relevance of iPLA2 in CCL3 signaling was further corroborated in experiments, in which iPLA2 expression was successfully (Figures 6(b), 6(c), and 6(d)) silenced by transfection of specific siRNA (*p* = 0.029, *n* = 4; Figure 6(e)). Taken together, these results are in line with the hypothesis that iPLA2 is involved in CCL3 signaling and might be a key enzyme for the production of the bioactive soluble factor(s) that presumably stimulate nAChR.

### 3.7. Limitations of the Study

This study has several limitations, and certainly, more research is needed to corroborate the data presented and to substantiate the intriguing concept that chemokines attracting monocytes inhibit ATP-mediated inflammasome activation. Here, we only investigated chemokines CCL3, CCL4, and CCL5 with a focus on CCL3. The concept should be confirmed with other relevant chemokines that also attract monocytes. Further, we used the monocytic cell line U937 as a model for human blood monocytes in most experiments. These cells were chosen as they possess the cholinergic control mechanism of IL-1*β* release typical for human blood monocytes [18, 20]. U937 cells, however, secrete very low amounts of IL-1*β* in response to ATP in comparison to primary human and mouse cells [18, 20]. Therefore, we corroborated the inhibitory potential of CCL3 also for primary human PBMC in vitro. In the same line, the concept should be tested in vivo, which will be very difficult due to the high redundancy of chemokines and chemokine receptors [12, 13, 15]. In addition, there are numerous open questions regarding the details of the proposed novel signal transduction mechanism, including the identification of the bioactive soluble factor(s) activating nAChR.

### 3.8. Biological and Clinical Relevance

Inflammasome activation, resulting in the production of inflammasome-dependent mediators and monocyte/macrophage pyroptosis, is a double-edged sword that is required for host defense against infections but is associated with the risk of inducing life-threatening SIRS [1–3, 11]. The benefits and risks of IL-1*β* probably depend on the anatomical compartment to which this mediator is released. Release of monocytic Il-1*β* to the blood stream is expected to be of limited use as the cytokine is swept away from the site of inflammation, but in contrast, the risk of inducing harmful systemic inflammation is high. In this study, we demonstrate that chemokines block ATP-dependent IL-1*β* release by blood monocytes, a mechanism that would prevent systemic inflammation. In contrast, release of IL-1*β* by inflammatory macrophages within the tissue fights against local infections and certainly causes less systemic effects. We expect that IL-1*β* can be released to inflamed tissue by macrophages in despite of high local chemokine levels, because pathogens induce inflammasome activation by several ATP-independent mechanisms that are probably not sensitive to chemokines [2, 6, 8, 9]. In addition, it might be hypothesized that chemokines do not inhibit the ATP-dependent IL-1*β* release of tissue macrophages, a topic that certainly deserves more investigation.

## 4. Conclusions

**Figure 7:**
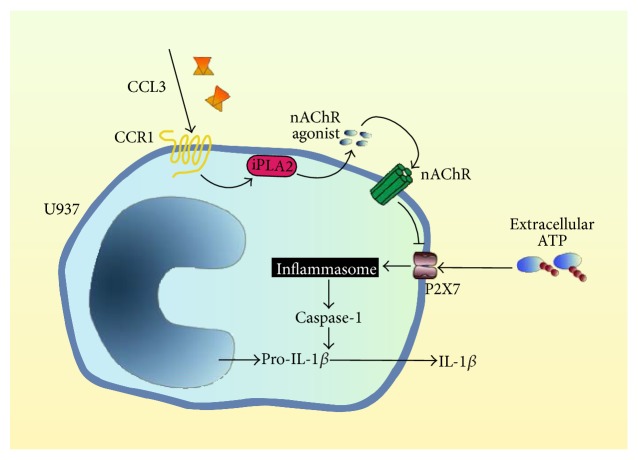
Schematic presentation of the proposed mechanism. The binding of extracellular ATP to P2X7 receptor on LPS-primed human monocytic U937 cells results in formation of a multiprotein complex called inflammasome that, in turn, activates caspase-1. Caspase-1 catalyzes the proteolytic maturation of pro-IL-1*β* and enables the release of mature, bioactive IL-1*β*. We propose that when U937 cells are stimulated with ATP and chemokine CCL3 concomitantly, chemokine receptor CCR1 activates the calcium-independent phospholipase A2 (iPLA2) and the secretion of a small agonist of nicotinic acetylcholine receptors (nAChR). Stimulation of nAChR containing subunits *α*7 and *α*9 inhibits P2X7 receptor function and, hence, maturation and secretion of IL-1*β*. It is still unclear if nAChR subunits of monocytic cells actually form conventional pentamers as shown in the schematic drawing.

Our results are in line with the hypothesis that CCL3, CCL4, and CCL5 inhibit BzATP-induced maturation and release of IL-1*β* by LPS-primed monocytic cells. CCL signaling seems to depend on binding to cognate CCR, activation of iPLA2, and release of soluble agonists of nAChR containing subunits *α*7 and *α*9 that inhibit IL-1*β* release (Figure 7). It has been shown before that activation of monocytic nAChR is a potent way to inhibit ATP-induced activation of P2X7 receptor, inflammasome activation, and release of IL-1*β* [18, 20]. The control of inflammasome activation in despite of the presence of ATP is of outstanding clinical interest. A better understanding of the underlying mechanisms might lead to the development of therapeutic strategies for the prevention and treatment of inflammatory diseases. With all due caution, we suggest a novel CCL-induced anti-inflammatory triple-membrane-passing signaling pathway inhibiting premature inflammasome activation in monocytes in response to extracellular ATP.

This mechanism might reduce trauma-induced release of IL-1*β* into the circulation and thereby prevent sterile SIRS. As trauma is often associated with infection, infiltrating monocytes/macrophages and local IL-1*β* release at the site of inflammation are desirable. As PAMP-induced inflammasome activation is typically ATP independent [9], local secretion of inflammasome-dependent cytokines by infiltrating monocytes/macrophages should be enabled, in despite of the presence of chemokines.

## Figures and Tables

**Table 1:** List of primers used for real-time RT-PCR.

Gene name (accession number)	Forward primer 5′-3′	Reverse primer 5′-3′	Amplicon size (bp)
*CCR1* (NM_001295.2)	GGA CAA AGT CCC TTG GAA CC	GGA GTT GCA TCC CCA TAG TC	101
*CCR3* (NM_178328.1)	ATC CGG GCA AGA ACT TAT CG	AGG ATG TGG TAC CAA AGG TCT C	114
*CCR5* (NM_000579.3)	CTG GCC AGA AGA GCT GAG AC	GGG CTC CGA TGT ATA ATA ATT GA	116
*CXCR6* (NM_006564.1)	GGA ACA AAC TGG CAA AGC AT	TGG CTG CTG TCA TTG AAA CT	107
*PLA2G6* (NM_003560.2)	CAT CCG TAA CCA CCC CAG C	CGT TCT CCG CGC AAT TGG	109

*CCR1*: C-C motif chemokine receptor 1; *CCR3*: C-C motif chemokine receptor 3; *CCR5*: C-C motif chemokine receptor 5; *CXCR6*: C-X-C chemokine receptor type 6; *PLA2G6*: phospholipase A2 group VI.
